# A critical assessment of microbial-based antimicrobial sanitizing of inanimate surfaces in healthcare settings

**DOI:** 10.3389/fmicb.2024.1412269

**Published:** 2024-06-12

**Authors:** Sabina Fijan, Peter Kürti, Urška Rozman, Sonja Šostar Turk

**Affiliations:** ^1^Faculty of Health Sciences, University of Maribor, Maribor, Slovenia; ^2^SMV-Probiotics, København, Denmark

**Keywords:** microbial cleaning, hygiene, *Bacillus*, spores, beneficial microbes, *genus Priestia*

## Abstract

The global rise in antimicrobial resistance (AMR) poses a significant public health threat, especially in healthcare settings, where controlling the spread of antimicrobial genes is crucial. While person-to-person transmission remains the primary route for healthcare-associated infections (HAIs), hospital surfaces serve as key reservoirs for antimicrobial-resistant microorganisms. Regular cleaning and disinfection of these surfaces are essential. Microbial-based products for sanitizing hospital surfaces have emerged as promising tools to combat HAIs and AMR. However, a review of 32 publications found inconsistencies and potential risks. A total of 15 publications included hospital-based trials, while the rest were either *in vitro* or *in situ* assays, reviews, book chapters, or commentaries. In most of the hospital-based studies, specific strains of applied microorganisms were not identified, and the term “probiotic” was inaccurately used. These products mainly featured spores from *Bacillus* and *Priestia* genera, which was mainly hypothesized to work through competitive exclusion. Most hospital-based studies have shown that the application of microbial-based products resulted in a significant reduction in pathogens on surfaces, thereby contributing to a decrease in the incidence of healthcare-associated infections (HAIs). Further research is however needed to understand the effectiveness, mechanisms of action, and safety of microbial-based sanitizing agents. Strain-level identification is crucial for safety assessments, yet many reviewed products lacked this information. Consequently, there is a need for rigorous safety evaluations within existing regulatory frameworks to ensure the efficacy and safety of microbial-based cleaning products in healthcare settings.

## Introduction and research aim

In healthcare settings, pathogenic and potentially pathogenic microorganisms can survive on hard surfaces for periods of up to several weeks (Wilks et al., 2005; Fijan et al., 2017). Transmission of microorganisms can occur between patients, healthcare professionals, and visitors (Fijan et al., 2005; Huslage et al., 2010; Fijan and Turk, 2012; Otter et al., 2013; Dancer, 2014; Sood and Perl, 2016; Suleyman et al., 2018). Hospital-associated infections (HAIs) are tightly associated microorganisms carrying antimicrobial resistance (AMR) genes. Opportunistic pathogens such as *Acinetobacter baumannii, Staphylococcus epidermidis, Enterococcus faecalis, Enterococcus faecium, Klebsiella pneumoniae, Pseudomonas aeruginosa, Staphylococcus aureus, Escherichia coli, Proteus mirabilis, Clostridioides difficile, Candida,* and *Aspergillus* spp. have been found on various hospital surfaces and environments and are especially hazardous for immunosuppressed or immunocompromised patients (Protano et al., 2019; Christoff et al., 2020; Asma et al., 2023). This was also evident during the recent COVID-19 epidemic, where the nature and extent of hospital contamination indicated that SARS-CoV-2 is likely dispersed conjointly through several transmission routes, including short- and long-range aerosol, droplet, and inanimate transmission (Ribaric et al., 2022).

The main interventions for reducing HAIs, especially in hospital settings, include environmental disinfection, antimicrobial stewardship, and hand hygiene (Louh et al., 2017). For the control and prevention of healthcare-associated infections, cleaning, disinfection, or sterilization in hospital environment is a well-known and established process (Rutala, 1996; McDonnell and Russell, 1999). Disinfectants containing one or more biocidal active substances (ECHA, 2023) are essential tools in combatting the spread of infectious diseases. However, overuse and improper use of disinfectants can affect resistance against disinfectants (Rutala and Weber, 2004).

Antimicrobial resistance (AMR) is a major healthcare concern worldwide. According to the World Health Organization, ESKAPEE group of pathogens (*Enterococcus faecium*, *Staphylococcus aureus, Klebsiella pneumoniae, Acinetobacter baumannii, Pseudomonas aeruginosa*, *Enterobacter* spp., and *Escherichia coli*) frequently exhibit multidrug resistance (Mulani et al., 2019; Kaiser et al., 2023; Neidhöfer et al., 2023). The rise in antibiotic resistance and the recent pandemic added new impetus to research in the field of cleaning and hygiene and the development of new and alternative antimicrobials (Willing et al., 2018) and cleaning products.

As an emerging category, microbial-cleaning products containing live bacteria or viable spores are being commercialized also for hospital use. The most common microbial components are spore former *Bacillus* spp. Spores are robust, metabolically inert, and resilient structures that are resistant to environmental stresses, such as UV, desiccation, heat, disinfectants, high temperatures, and antibiotics (Buddle and Fagan, 2023; Fijan et al., 2023). *Bacillus* spp. are used in household cleaners, personal care products, animal hygiene or garden-related cleaning applications, face mask sanitizing sprays, air conditioner-cleaning products, a waterbed conditioner, and drain-unblocking products, and all are subjected to specific authorization protocols. As an example, European Union sanitizing products may be subjected to regulations for cleaning agents, detergents, personal care products, cosmetics, or biocide regulations (EUR-Lex, 2012; Razenberg et al., 2020). Because of the microbial component, uncertainty prevailed about the regulatory category for microbial-cleaning products. Although microbial-based products are advertised as being safe for humans, no third-party safety assessment ascertains these claims. Microorganisms added to microbial-cleaning products may pose a risk to humans, animals, and the environment, especially in hospital settings. The EU microbial-cleaning products are subjected to biocide regulation which requires strain level safety assessment.

Currently, only one bacterial strain is approved as biocide for veterinary use (Razenberg et al., 2020). Hence, a significant finding of this review is that none of the microbial-cleaning products—which are tested in hospital settings within the European Union—has undergone safety assessment by competent authorities within the framework of Biocidal Products Regulation.

This review aims to assess the effect of microbial-cleaning products and emphasize the importance of strain information, safety, the presence of antimicrobial resistance, and mechanisms of action of microbial-based cleaning products when used in hospital settings.

## Microbes and their competitive and antagonistic strategies

**Figure 1:**
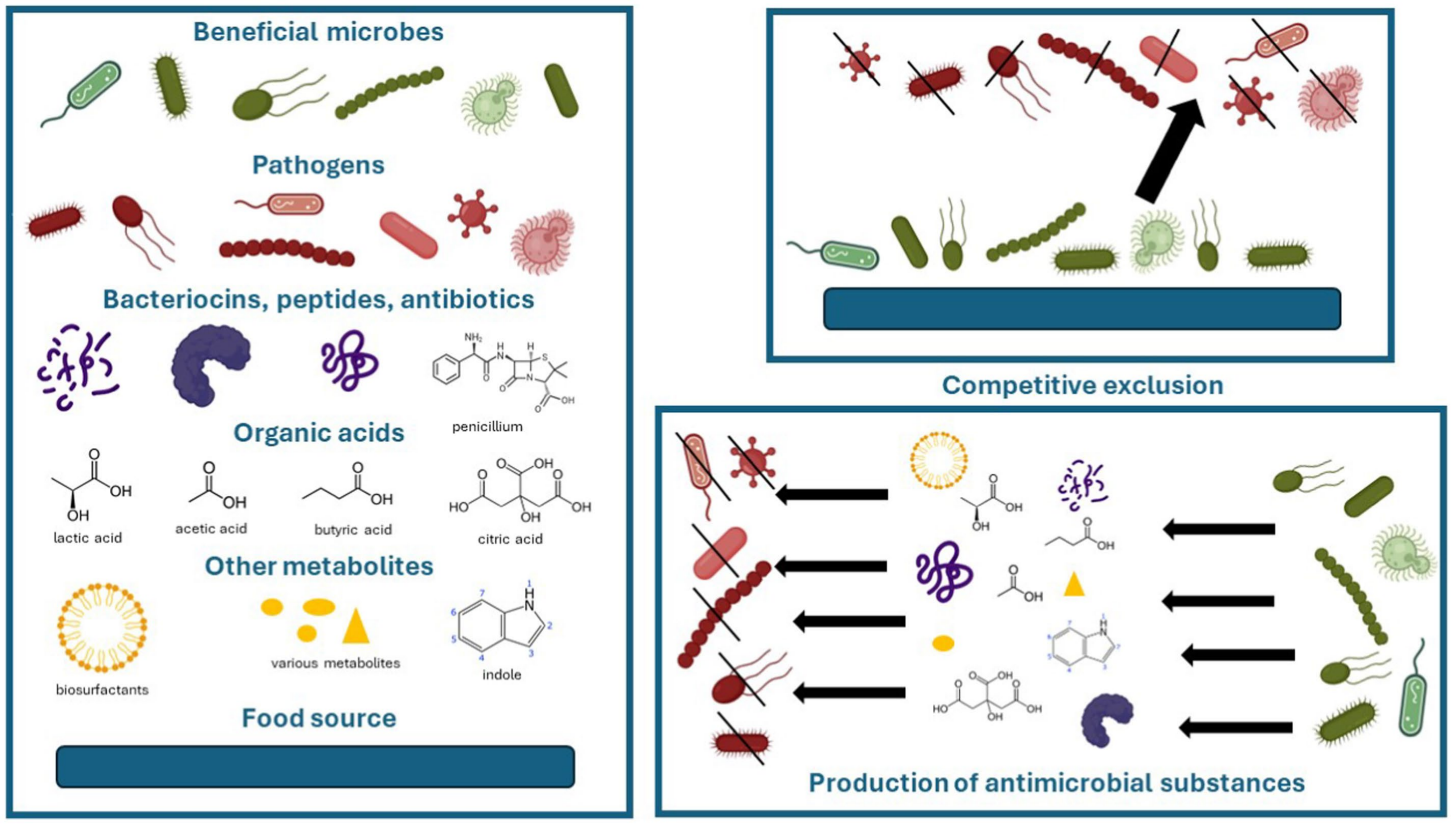
Competitive and antagonistic strategies of beneficial microbes against pathogens. Images of microbes and peptides were accessed from BioRender.com.

Microorganisms are microscopic organisms that mainly consist of bacteria, archaea, fungi, protists, (protozoa and algae), and viruses (Sattley and Madigan, 2015). In relation to other organisms, they can be pathogens, commensals, or beneficial. In the environment, they can compete for nutrients to survive. Microorganisms have various antagonistic strategies to outcompete or inhibit each other such as competitive exclusion and production of antimicrobial substances. Competitive exclusion states that two species that use similar resources cannot coexist indefinitely in the same ecological niche (Kneitel, 2008), especially if nutrients are scarce. Antimicrobial substances are a variety of chemical or physical substances that inhibit or destroy another organism, such as antibiotics, bacteriocins or antimicrobial peptides, biosurfactants, organic acids, and many others (Moody and Needles, 2004; Fijan, 2014; Agarwal et al., 2016; Engevik Melinda and Versalovic, 2017; Huang et al., 2021; Mercado and Olmos, 2022; Zhang et al., 2022). Well-known bacteriocins include colicins, nisins, microcins, and lantibiotics (Cotter et al., 2013). Organic acids produced by microbes include lactic acid, acetic acid, propionic acid, butyric acid, citric acid, succinic acid, and (Sauer et al., 2008; Chen and Vitetta, 2020; Punia Bangar et al., 2022) indole. By production of these antimicrobial substances, one group of microorganisms can prevent the growth of other microorganisms. The key objects of action are the destruction of cell walls, cytoplasmic membranes, and inhibition of multiplication that leads to the death of microorganisms (Surendran Nair et al., 2017; Punia Bangar et al., 2022). These strategies can also be utilized by beneficial microbes against pathogens and are presented in Figure 1.

## Healthcare settings and environment microbiome

Falagas and Makris (2009) proposed the concept of ‘environmental microorganisms’ with the objective of inhibiting the growth of nosocomial pathogens on inanimate surfaces and being a safe and effective intervention for infection control. It was suggested that environmental microorganisms could be applied to patient care equipment, e.g., tubes and catheters with the aim of reducing nosocomial pathogen colonization. Notably, as we discuss later, a probiotic definition is inappropriately used for this application.

According to Protano et al. (2019) the indoor ‘healthcare building environment microbiome‘(D’Accolti et al., 2022) should be predominantly composed of commensal and beneficial microbes. However, the hospital microbiome also contains human pathogens, including pathogens with anti-microbial resistance genes, which originated from infected individuals. The building microbiome is very diverse and dynamic; therefore, understanding indoor microbiome can result in novel solutions to reduce hospital acquired infections (Cason et al., 2022; D’Accolti et al., 2022).

## The inappropriateness of labeling microbial-based sanitizing products as probiotics

Probiotics are by definition ‘live microorganisms that, when administered in adequate amounts, confer a health benefit on the host’ (Hill et al., 2014). This definition is a product of scientific discussions and is generally accepted by stakeholders of probiotic science and related industries. The definition underlines that only beneficial microbes with a scientifically proven health effect on humans or animals are probiotics. Because of intra-species genetic diversity, the probiotic health claims are strain-related and cannot be generalized to a whole species. For evidencing a health claim, minimum one human clinical study is required using a defined microbial strain or combination of strains. The health outcome must be statistically significant when compared with a control group. Whatsoever beneficial effects of spreading microbes on environmental surfaces are not probiotic effects. The health effects of orally taken probiotics by no means can be translated to any desired effect on abiotic surfaces in hospital buildings when used as a microbial cleaning product. One example is *Escherichia coli* Nissle 1917, which is a well-known probiotic with health benefits established via gut microbiota modulation (Petersen et al., 2014; Manzhalii et al., 2016; Scaldaferri et al., 2016). However, it is ineffective when used in other microbial habitats of the human body, for example, the vaginal microbiome or urobiome (Buchta, 2018; Jones et al., 2021). On inanimate surfaces, the use of this strain is useless. As to the legal aspects, the European Food Safety Authority (EFSA) considers the term probiotic as an unauthorized health claim that may not be used in food labeling (Hill and Sanders, 2013; Sanders et al., 2014). It remains a question whether such scrutiny is also adequate for the labeling of microbial-cleaning products.

The mechanisms of action of probiotics are well-known and encompass several key aspects, such as competitive exclusion, modulation of the microbiome, excretion of diverse antimicrobial compounds (including bacteriocins, organic acids, and other antimicrobial compounds), immunomodulation, and interaction through various gut-organ axes (Zhou and Foster, 2015; Salem et al., 2018; Fijan et al., 2019; Maldonado Galdeano et al., 2019). It is important to note that many of these characteristics are host-specific and cannot be universally applied to all hosts, body sites, and inanimate surfaces.

## Search strategy and results of literature overview

A literature overview was conducted to summarize the existing studies of using microbial-based cleaning agents for inanimate surfaces in healthcare settings. We used the search strategy: (“microbial wipes” OR “microbial cleaning” OR “probiotics”) AND (“healthcare” OR “hospital”) AND (“cleaning” OR “disinfection”) in various databases (PubMed, ScienceDirect, and manual search). Publications in languages other than English without adequate information were excluded. A total of 32 publications were found on the selected subject/topic (up to 10th of May 2024). For each publication, data about author(s), publication year, aim, microorganisms with antimicrobial effect, and main findings were extracted. The results are presented in Table 1 in descriptive form.

**Table 1:** Information on 32 publications using microbial-based antimicrobial sanitizing of hospital settings, listed in ascending chronological and alphabetical order.

	Reference^#^	Publication type	Aim	Microorganisms in sanitizing product or for antagonism against pathogens.	Main findings
1	Falagas and Makris (2009)	Opinion	Antagonistic effects of cleaning product on non-biological surfaces.	*Streptococcus thermophilus* A, *Lactococcus lactis* 53, *Streptococcus mitis* BA and BMS, *Lactobacillus acidophilus* RC 14 and undefined lactobacilli.	Decreased adhesion of selected pathogens on non-biological surfaces after using cleaning product.
2	Manning et al. (2009)	Patent	Antibacterial effect of liquid cleaning agent against pathogens on surfaces.	Product with undefined strains of *Bacillus, Pseudomonas, Arthrobacter, Enterobacter, Citrobacter* and *Corynebacterium*. Other components.	Effectiveness of antibacterial cleaning agent against various selected hospital pathogens.
3	Kohli (2013)	Book chapter	Effect of cleaning agent against HAI-related pathogens on surfaces.	Products with undefined strains of *Bacillus, Pseudomonas*.	Effectiveness of antibacterial cleaning agent against various hospital pathogens based on reference (Manning et al., 2009).
4	Vandini et al. (2014a)	*In vitro* and *in situ* assays	Effect of a PCHS^®^ against surface contamination.	Microbial-based cleaning product (PCHS^®^) with undefined strains of *Bacillus subtilis, Bacillus pumilus* and Priestia megaterium* (3×10^7^ cfu/mL).	Reduction of pathogenic bacteria on contaminated surfaces.
5	Vandini et al. (2014b)	Multi-centric hospital-based study	Effect of PCHS^®^ against HAI-related pathogens on hospital surfaces.	Microbial-based cleaning product (PCHS^®^) with undefined strains of *Bacillus subtilis, Bacillus pumilus* and Priestia megaterium* (5×10^7^ cfu/mL).	Reduction of pathogens on hospital surfaces after cleaning with PCHS^®^. No mutagenicity or gene transfer events, even 12 months after application.
6	Arvanitakis (2015)	Chapter –Review	Use of microorganisms in antimicrobial cleaning products.	*Bacillus subtilis, Bacillus licheniformis, Bacillus amyloliquefaciens, Bacillus polymyxa, Lactobacillus* sp., *Saccharomyces cerevisiae*.	Significant gaps in the knowledge of cleaning products and the specific strains used as the active ingredients.
7	La Fauci et al. (2015)	*In vitro* and hospital-based study	*In vitro* analysis and hospital trial on the effectiveness of PCHS^®^ against surface contamination.	Microbial-based cleaning product PCHS^®^ with undefined strains of *Bacillus subtilis, Bacillus pumilus* and Priestia megaterium* spores (3×10^7^ cfu/mL) and additional components.	Average reduction of pathogens up to 99.9% on surfaces with *in vitro* tests. Reduction of pathogens on hospital surfaces after cleaning with PCHS^®^.
8	Caselli et al. (2016)	Hospital-based study	Effect of PCHS^®^ against HAI-related pathogens on hospital surfaces and impact on drug resistance.	Microbial-based cleaning product PCHS^®^ containing spores of unidentified strains of Bacillus subtilis, Priestia megaterium* and *Bacillus pumilus* spores (10^7^ cfu/mL).	*Bacillus* spores germinated on dry inanimate surfaces and counteracted the growth of pathogens. Decrease of antibiotic resistance genes in the microbial population.
9	Afinogenova et al. (2018)	Hospital-based study	Effect of microbial-based cleaning at treatment room.	Microbial-based cleaning containing spores of unidentified strains of *Bacillus subtilis, Priestia licheniformis* and *Bacillus pumilus* spores. cfu-nd.	Inhibition of growth and proliferation of sanitary-indicative microorganisms.
10	Al-Marzooq et al. (2018)	Hospital-based study	Effect of microbial-based cleaning in a dental clinic.	Undefined strain of *Bacillus subtilis*, concentration: not mentioned.	Decrease of surface pathogens in a dental clinic using of microbial-based sanitation.
11	Rodolfi and Caselli (2018)	Patent	Product for cleaning and sanitizing surfaces	Microbial-based cleaning product PCHS^®^ containing spores of unidentified strains of Bacillus subtilis, Priestia megaterium* and *Bacillus pumilus* and bacteriophages and additional components.	Decreased adhesion of selected pathogens on non-biological surfaces. Presence of bacteriophages increases effectiveness.
12	Caselli et al. (2018)	Multi-centric hospital-based study	Effect of PCHS^®^ on surface contamination and HAI-related incidence in hospitalized patients.	Microbial-based cleaning product PCHS^®^, containing spores of unidentified strains of Bacillus subtilis, Priestia megaterium* and *Bacillus pumilus*.	Significant decrease of HAI cumulative incidence in hospitalized patients and stable decrease of investigated surface pathogens after cleaning with PCHS^®^.
13	D'Accolti et al. (2018)	*In vitro* and *in situ* assays	Effect of a combined use of phages and PCHS^®^ in removing HAI-related pathogens.	Combined use of microbial-based cleaning product PCHS^®^ containing spores of unidentified strains of *Bacillus subtilis, Bacillus pumilus* and Priestia megaterium* and bacteriophages (10^5^–10^6^ PFU/mL).	Reduction of the investigated pathogens after cleaning with PCHS^®^ and phages. *In situ* assay also resulted in reduction of inoculated pathogen on surfaces.
14	Al Marzooq et al. (2019)	Conference abstract	Effect of microbial-based cleaning in a dental clinic.	Undefined strain of *Bacillus subtilis*.	Same as Al-Marzooq et al. (2018)
15	Caselli et al. (2019)	Multi-centric hospital-based study	Effect of PCHS^®^ on antimicrobial resistance and HAI-related antimicrobial consumption.	Microbial-based cleaning product PCHS^®^ containing spores of unidentified strains of *Bacillus subtilis, Bacillus pumilus* and Priestia megaterium*.	Decrease of AMR genes, harbored by surface hospital microbiota after use of PCHS^®^. Decrease of antimicrobial drug consumption associated with HAI.
16	D'Accolti et al. (2019)	Hospital-based study	effect of a combined use of phages and PCHS^®^ in reducing staphylococci contamination on hospital surfaces.	Microbial-based cleaning product PCHS^®^ containing spores of unidentified strains of *Bacillus subtilis, Bacillus pumilus* and Priestia megaterium* combined with bacteriophages (2×10^8^ PFU/mL).	Reduction of staphylococci load on treated hospital surfaces after use of PCHS^®^ and phages.
17	Dural-Erem et al. (2019)	*In vitro* assays	Viability of spores on wipes for surface cleaning.	Tana^®^ Biotic DC with undefined strains of *Bacillus* spores with additional component.	Adequate number of spores of Tana^®^ Biotic DC after wetting grew and colonized on the wiped surfaces.
18	Kleintjes et al. (2019)	Hospital-based study	Effect of microbial-based cleaning in a burn unit.	Microbial-based cleaning product containing spores of unidentified strains of Bacillus subtilis, Bacillus pumilus* and *Priestia megaterium*.	Microbial-based cleaning agent did not significantly reduce the colonization of pathogens.
19	Rognoni et al. (2019)	Conference abstract	Economic impact of the HAI-related infection management with PCHS^®^.	Microbial-based cleaning product PCHS^®^ containing spores of unidentified strains of *Bacillus subtilis, Bacillus pumilus* and Priestia megaterium*.	Poster abstract based on the results of Caselli et al. (2018, 2019).
20	Caselli and Purificato (2020)	Commentary	Commentary on the results of recently published study.	Microbial-based cleaning product PCHS^®^, containing spores of unidentified strains of *Bacillus subtilis, Bacillus pumilus* and Priestia megaterium*.	Commentary based on the results of Caselli et al. (2018, 2019).
21	Kleintjes et al. (2020)	Hospital-based study	Long-term effect of microbial-based cleaning in a burn unit.	Microbial-based cleaning product containing spores of unidentified strains of Bacillus subtilis, Bacillus pumilus* and *Priestia megaterium*.	Retrospective analysis of HAI after use of microbial-based cleaning agent showed reduction of pathogens.
22	Razenberg et al. (2020)	Report	Review of microbial cleaning products on the Dutch market.	Various undefined strains of the *Bacillus* genus.	92 different microbial cleaning products with often incomplete composition and regulatory information.
23	Stone et al. (2020)	*In vitro* assay	Effect of surface cleaning products on the resident microbiome.	A patented *Bacillus* spore consortium.	The microbiome established on surfaces using microbial-based cleaning did not completely outcompete the pathogens.
24	Tarricone et al. (2020)	Multi-centric hospital-based study	Impact of lower HAI incidence after use of PCHS^®^.	Microbial based cleaning product PCHS^®^, containing spores of unidentified strains of *Bacillus subtilis, Bacillus pumilus* and Priestia megaterium*.	Lower incidence of HAI, severe HAI and antibiotic resistance after use of PCHS in hospitals.
25	D’Accolti et al. (2021)	*In vitro* assay	Assessment of antiviral properties of PCHS^®^ against enveloped viruses.	Microbial based cleaning product PCHS^®^, containing spores of unidentified strains of *Bacillus subtilis, Bacillus pumilus* and Priestia megaterium*.	Inactivation of enveloped viruses on treated hospital surfaces after use of PCHS^®^.
26	D’Accolti et al. (2022)	Review	Role of cleaning and disinfecting of healthcare surfaces.	Microbial based cleaning product PCHS^®^, containing spores of unidentified strains of *Bacillus subtilis, Bacillus pumilus* and Priestia megaterium*.	Stabilization of hospital environmental microbiome to lower the concentration of pathogens.
27	Klassert et al. (2022)	Multi-centric hospital-based study	Effect of sanitizing with *Bacillus* spp. containing detergents on the environmental microbiome.	Product containing *Bacillus subtilis* ATCC 6051, Priestia megaterium* ATCC 14581, *B. licheniformis* ATCC 12713, *B. amyloliquefaciens* DSL 13563-0, *B. pumilus* ATCC 14884 and additional components.	Displacement of intrinsic environmental microbiota after cleaning with microbial based solution.
28	Soffritti et al. (2022)	Hospital-based study	Effect of PCHS^®^ in the emergency room of a children’s hospital.	Microbial based cleaning product PCHS^®^, containing spores of unidentified strains of *Bacillus subtilis, Bacillus pumilus* and Priestia megaterium*.	Stable decrease in surface pathogens and resistance after use of PCHS^®^.
29	Caselli (2023)	Conference abstract	Effect of PCHS^®^	Microbial based cleaning product PCHS^®^, containing spores of unidentified strains of *Bacillus subtilis, Bacillus pumilus* and Priestia megaterium*.	Presentation of results on using PCHS^®^.
30	D’Accolti et al. (2023)	Multi-centric hospital-based study	Effect of PCHS^®^ added specific anti-staphylococcal phage on pathogen.	PCHS^®^, containing spores of unidentified strains of Bacillus subtilis, Priestia megaterium* *and Bacillus pumilus* with added phages.	Removal of staphylococci after use of PCHS^®^ with added specific anti-staphylococcal phage.
31	Leistner et al. (2023)	Multi-centric hospital-based study	Effect of three different surface-cleaning strategies on the incidence of HAIs.	SYNBIO^®^ containing *Bacillus subtilis* ATCC 6051, Priestia megaterium* ATCC 14581, *B. licheniformis* ATCC 12713, *B. amyloliquefaciens* DSL 13563-0, *B. pumilus* ATCC 14884 (3×10^7^ cfu/mL).	Surface disinfection using SYNBIO^®^ was not superior to soap-based cleaning or disinfection in terms of HAI.
32	Ramos and Frantz (2023)	Review	Effect of sanitation of healthcare surfaces.	Various microbial based cleaning products, containing *Bacillus* spp.	Reduction in pathogen burden and nosocomial infections using microbial-based solutions.

^#^First author and year of publication; *Priestia megaterium (previously *Bacillus megaterium*) (Gupta et al., 2020); HAI, healthcare associated infections; cfu-nd, no data on cfu of microbial-based product; PFU, plaque forming units.

The 32 publications noted in Table 1 (Falagas and Makris, 2009; Manning et al., 2009; Kohli, 2013; Vandini et al., 2014a,b; Arvanitakis, 2015; La Fauci et al., 2015; Caselli et al., 2016; Afinogenova et al., 2018; Al-Marzooq et al., 2018, 2019; Caselli et al., 2018; D'Accolti et al., 2018, 2019, 2021, 2022, 2023; Rodolfi and Caselli, 2018; Caselli et al., 2019; Dural-Erem et al., 2019; Kleintjes et al., 2019; Rognoni et al., 2019; Caselli and Purificato, 2020; Kleintjes et al., 2020; Razenberg et al., 2020; Stone et al., 2020; Tarricone et al., 2020; Klassert et al., 2022; Soffritti et al., 2022; Caselli, 2023; Leistner et al., 2023; Ramos and Frantz, 2023) include an opinion (Falagas and Makris, 2009), two patents (Manning et al., 2009; Rodolfi and Caselli, 2018), a commentary (Caselli and Purificato, 2020), a report (Razenberg et al., 2020), two reviews (D’Accolti et al., 2022; Ramos and Frantz, 2023), two book chapters (Kohli, 2013; Arvanitakis, 2015), three conference abstracts of previously published results (Al Marzooq et al., 2019; Rognoni et al., 2019; Caselli, 2023), and *in vitro* assays and hospital-based studies. A total of five publications assessed the efficiency of microbial-based sanitization products using *in vitro* assays with common healthcare-associated pathogens (La Fauci et al., 2015; D'Accolti et al., 2018, 2021; Dural-Erem et al., 2019; Stone et al., 2020). Among these studies, two were *in situ* assays (D'Accolti et al., 2018) and one was a hospital-based study (La Fauci et al., 2015). A total of 15 publications presented hospital-based studies (Vandini et al., 2014b; La Fauci et al., 2015; Caselli et al., 2016, 2018, 2019; Al-Marzooq et al., 2018; D'Accolti et al., 2019, 2023; Tarricone et al., 2020; Klassert et al., 2022; Soffritti et al., 2022; Leistner et al., 2023). Of these, 7 were multicentric studies (Vandini et al., 2014b; Caselli et al., 2018, 2019; Tarricone et al., 2020; Klassert et al., 2022; D’Accolti et al., 2023; Leistner et al., 2023) and 8 were monocentric (La Fauci et al., 2015; Caselli et al., 2016; Afinogenova et al., 2018; Al-Marzooq et al., 2018; D'Accolti et al., 2019; Kleintjes et al., 2019, 2020; Soffritti et al., 2022). One of these publications included an *in vitro* assay (La Fauci et al., 2015). Several of these hospital-based studies used the term randomized controlled trial.

To avoid confusion, we used the term hospital-based study in Table 1 for all intervention studies that were conducted in hospitals and investigated either the direct use of microbial-based products on hospital surfaces (the reduction in the concentration of causative agents of HAIs on the treated surfaces) or the indirect use of the microbial-based products (the reduction in the incidence of HAIs among the hospitalized patients after treatment of hospital surfaces) (Vandini et al., 2014b; La Fauci et al., 2015; Caselli et al., 2016, 2018, 2019; Al-Marzooq et al., 2018; D'Accolti et al., 2019, 2023; Tarricone et al., 2020; Klassert et al., 2022; Soffritti et al., 2022; Leistner et al., 2023).

In the 32 studies, the most prevalent microbes used for the microbial-based products were spore formers of the genus *Bacillus*. However, the genus *Bacillus* has recently been further divided (Gupta et al., 2020) and reclassified (Narsing Rao et al., 2023). For example: *Bacillus megaterium* has been reclassified as *Priestia megaterium, Bacillus sphaericus* to *Lysinibacillus sphaericus,* and *Bacillus coagulans* to *Heyndrickxia coagulans*. Since species and strain information were not available in most of the studies, it is impossible to correctly note which genera were used. Correct and up-to-date genera, species, and strain designation are therefore imperative for future research of the category.

‘Probiotic Cleaning Hygiene System’ (PCHS®) was first mentioned in the publication by Vandini et al. (2014a) as a product manufactured by Chrisal (Lommel, Belgium). It contains undefined strains of *Bacillus subtilis, Bacillus pumilus,* and *Priestia megaterium* (previously *Bacillus megaterium*). Sixteen subsequent publications either refer to this product or mention a previous publication that referred to this product, without mentioning strain identity (Vandini et al., 2014b; La Fauci et al., 2015; Caselli et al., 2016, 2018, 2019; D'Accolti et al., 2018, 2019, 2021, 2022, 2023; Rodolfi and Caselli, 2018; Rognoni et al., 2019; Caselli and Purificato, 2020; Tarricone et al., 2020; Soffritti et al., 2022; Caselli, 2023). Further information from the publications noted that PCHS® was supplied by Copma srl (Ferrara, Italy). As a base solution, this product also contains non-ionic, cationic, and amphoteric surfactants. Among these publications that were *in vitro* assays to assess antimicrobial effects, trials were conducted in hospital or clinic settings, along with presentations and commentaries of results. Bacteriophages were added to PCHS®, as reported in three hospital-based studies (D'Accolti et al., 2018, 2019, 2023), one publication was a patent (Rodolfi and Caselli, 2018) and one was a review (D’Accolti et al., 2022). We contacted both companies about the product several times for a description of the strains used, but we did not receive an answer.

Two further studies also used unidentified strains of *Bacillus subtilis, Bacillus pumilus,* and *Priestia megaterium* (previously *Bacillus megaterium*) which were manufactured by Chrisal South Africa (supplied by Umsinsi Health Care) (Kleintjes et al., 2019, 2020). Tana® Biotic DC (Tanatex Chemicals, the Netherlands) contains undefined *Bacillus* strains, and polyethylene glycol was used in one study (Dural-Erem et al., 2019), and undefined strains of *Bacillus subtilis* from InnuScience, Canada, were used in a further study (Al Marzooq et al., 2019). The authors contacted both latter-mentioned companies for a description of their strains. Both requests were rejected for confidentiality reasons. Unidentified strains of *Bacillus subtilis, Bacillus licheniformis,* and *Bacillus pumilus* spores were used in a further study (Afinogenova et al., 2018).

Only two studies (Klassert et al., 2022; Leistner et al., 2023) declared strain identity in both studies, such as *Bacillus subtilis* ATCC 6051, *Priestia megaterium* ATCC 14581, *Bacillus licheniformis* ATCC 12713, *Bacillus amyloliquefaciens* DSL 13563-0, and *Bacillus pumilus* ATCC 14884. In the first study, the product was manufactured by Chrisal. The product also contains tensides, i.e., surfactants. The product tested in the latter publication is SYNBIO® (HeiQ Chrisal NV). Table 2 recaps the information on the microorganisms tested in the *in vitro*, *in situ,* and hospital-based studies.

**Table 2:** Information on microbial composition of microbial-based cleaning or sanitizing solutions and references of studies.

Microbial composition in microbial-based solutions	References
Bacillus subtilis* Bacillus pumilus* Priestia megaterium*	D’Accolti et al. (2022), Vandini et al. (2014a,b), La Fauci et al. (2015), Caselli et al. (2016), Rodolfi and Caselli (2018), Caselli et al. (2018), Caselli et al. (2019), Kleintjes et al. (2019), Rognoni et al. (2019), Kleintjes et al. (2020), Tarricone et al. (2020), D’Accolti et al. (2021), Soffritti et al. (2022), and Caselli (2023)
Bacillus subtilis* Bacillus pumilus* Priestia megaterium* bacteriophages	D'Accolti et al. (2018), D'Accolti et al. (2019), and D’Accolti et al. (2023)
*Bacillus* spp. **	Dural-Erem et al. (2019)
Bacillus subtilis*	Al Marzooq et al. (2019)
Bacillus subtilis* Bacillus licheniformis* Bacillus pumilus*	Afinogenova et al. (2018)
*Bacillus subtilis* ATCC 6051 *Priestia megaterium* ATCC 14581 *Bacillus licheniformis* ATCC 12713 *Bacillus amyloliquefaciens* DSL 13563-0 *Bacillus pumilus* ATCC 14884	Klassert et al. (2022) and Leistner et al. (2023)

* no strain information; ** no species information.

The most commonly monitored pathogens in the reviewed studies were ESKAPEE representatives, such as *Enterococcus faecium*, *Staphylococcus aureus, Klebsiella pneumoniae, Acinetobacter baumannii, Pseudomonas aeruginosa*, *Enterobacter* species, and *Escherichia coli*. Several studies (D'Accolti et al., 2018, 2022; Al Marzooq et al., 2019; Caselli and Purificato, 2020) investigated the survival of these pathogens on hospital surfaces or observed their incidence among hospital patients after microbial-based sanitation regimes.

## Main findings of studies investigating microbial-based antimicrobial sanitizing in hospital settings

Falagas and Makris (2009) conducted a review of the *in vitro* antagonistic studies of selected microorganisms against common pathogens, which were inoculated on various artificial non-biological surfaces. The selected microorganisms, researched for antagonism, included *Streptococcus thermophilus* A, *Lactococcus lactis* 53, *Streptococcus mitis* BA and BMS, *Lactobacillus acidophilus* RC 14, and undefined lactobacilli. Several investigated microorganisms decreased the adhesion of the selected pathogens (*Staphylococcus aureus, Staphylococcus epidermidis*, *Streptococcus mutans, Enterococcus faecalis, Candida albicans*, *Candida tropicalis, and Rothia dentocariosa*). They concluded that some of these organisms could represent a safe and effective intervention for infection control of environmental sites and medical devices in hospitals. The limitations of this study were the survival of organisms on non-biological surfaces and lack of antimicrobial effect against all pathogens.

Manning et al. (2009) applied for a patent for an antibacterial liquid-cleaning agent against various pathogens on surfaces. The product contained undefined strains of *Bacillus, Pseudomonas, Arthrobacter, Enterobacter, Citrobacter,* and *Corynebacterium* together with enzymes, surfactants, and an aqueous carrier. Patent viable microorganisms, which are capable of surviving in the intended environment, are incorporated in a cleaning composition, which was effective methicillin-resistant *Staphylococcus aureus*, vancomycin-resistant enterococci, glycopeptide-intermediate *Staphylococcus aureus,* and vancomycin-intermediate *Staphylococcus aureus*. The noted examples of applications include animal facilities and bioremediation. The patent status is ‘abandoned’. In the book chapter by Kohli (2013), information on the effectiveness of antimicrobial-cleaning agents containing undefined strains of *Bacillus* and *Pseudomonas* against HAI-related pathogens on surfaces was collected based on information from the previous reference (Manning et al., 2009). In the book chapter, Arvanitakis (2015) discussed the use of microbial strains, such as *Bacillus subtilis, Bacillus licheniformis, Bacillus amyloliquefaciens, Bacillus polymyxa, Lactobacillus* sp., and *Saccharomyces cerevisiae,* in antimicrobial-cleaning products. They found significant gaps in terms of what is known about the extent of commercial and domestic uses of these types of products and the specific strains of microorganisms used as the active ingredients. They suggested that genetically modified microorganisms could potentially play a significant role in the production of modified enzymes with enhanced properties for use as active ingredients in cleaning products for a variety of applications.

Vandini et al. published two studies in 2014. One was an *in vitro* and *in situ* assay (Vandini et al., 2014a) and the other one was a hospital-based study (Vandini et al., 2014b). Both used a microbial-based cleaning product named as PCHS® with undefined strains of *Bacillus subtilis, Bacillus pumilus, and Priestia megaterium*. In the first study (Vandini et al., 2014a), the effect of PCHS® in comparison with traditional disinfection treatment was assessed. *In vitro* assay found that the bacterial load of inoculated pathogens (*Escherichia coli, Staphylococcus aureus*, and *Pseudomonas aeruginosa*) on surfaces was reduced to a concentration of less than 10^2^ cfu. The *in situ* assays conducted in two different hospital settings in Italy also found a reduction, which was comparable to the conventional cleaning protocols. The muti-centric hospital-based study (Vandini et al., 2014b) was conducted in three hospitals (one in Belgium and two in Italy). The susceptibility and resistance tests of the microbes in the microbial cleaning agent PCHS® found no new or acquired resistance genes and no mutagenicity or gene transfer events, even 12 months after application. Some antimicrobial resistance of species, comparable to other *Bacillus* spp., was found. Cleaning hospital surfaces in three independent hospitals with PCHS resulted in a reduction of investigated pathogens, associated with HAI on surfaces by 50 to 89%, including isolates of coliforms and *Escherichia coli, Staphylococcus aureus*, *Clostridioides difficile*, and *Candida albicans* in 20,000 collected samples.

La Fauci et al. (2015) also investigated the effectiveness of PCHS® against surface contamination with HAI-related pathogens in a surgical ward and published the results of an *in vitro* and hospital-based study in 2015. This research noted that in addition to undefined strains of *Bacillus subtilis, Bacillus pumilus, and Priestia megaterium* spores, the product also contained non-ionic surfactants (0.6%), anionic surfactants (0.8%), and enzymes (amylases 0.02%). The *in vitro tests* using PCHS® found that an average reduction in investigated pathogens ranging from 92.2 to 99.9% on surfaces was achieved. The hospital trial in a surgical ward of an Italian hospital found 100% elimination of *Enterococcus faecalis* and *Candida albicans* from hospital surfaces after using PCHS followed by environmental sampling and almost 100% elimination of *Pseudomonas aeruginosa*, *Acinetobacter baumannii,* and *Klebsiella pneumoniae*, while results for the elimination of *Staphylococcus aureus* from hospital surfaces were lower.

Caselli et al. (2016) published the results of a hospital-based study in 2016 on the effect of PCHS® against surface contamination with HAI-related pathogens and the impact on drug resistance in a private hospital in Italy. Subspecies PCR characterization of PCHS® found all three species (*Bacillus subtilis, Priestia megaterium, and Bacillus pumilus*), but data are not shown. Environmental sampling after cleaning surfaces in the private hospital with PCHS® found that the *Bacillus* spores were germinated on dry inanimate surfaces, generating the bacterial vegetative forms which counteracted the growth of investigated pathogens by strongly decreasing their number and effectively substituting them on treated surfaces. The strongest evidence was found for *Staphylococcus aureus*. The procedure did not select resistant species but induced an evident decrease in antibiotic-resistant genes in the contaminating microbial population. Rodolfi and Caselli (2018) filed a patent application in 2016 for the microbial-based cleaning product PCHS® containing spores of unidentified strains of Bacillus subtilis, Priestia megaterium*, *and Bacillus pumilus* and non-ionic surfactants (5–15%), cationic surfactants (<5%), amphoteric surfactants (<5%), and bacteriophages. The patent was published in 2018 and granted in 2020. As a result of daily use for 30 days, the author found that *Staphylococcus aureus,* Enterobacteriaceae*, and Candida albicans* contamination was reduced by 90% when compared with other detergents. The bacteriophage component is comprised of combinations of the following order Caudovirales and/or families: Microviridae, Leviviridae, Inoviridae, Tectiviridae, or Corticoviridae. When used alone, the author found that the bacteriophage components eliminated 90% of contaminant microbes (10^2^ cfu/24 cm^2^) *in vitro* after 1 h of exposure. The combined use of PCHS® and bacteriophages resulted in almost total decontamination from selected pathogens (*S. aureus, P. aeruginosa,and C. albicans*) after continuous exposure for 4 weeks.

The antibacterial effectiveness of microbial-based cleaning in a dental clinic in UAE using undefined strains of *Bacillus subtilis* (Innu Science, Canada) was assessed by Al-Marzooq et al. (2018). They found that the product had a stronger effect on surface pathogens in the dental clinic compared with conventional disinfectants. Bacterial counts of staphylococci, streptococci, and gram-negative rods were significantly reduced from almost all treated surfaces of the dental clinic. The results were also presented at a conference in 2019 (Al Marzooq et al., 2019).

Caselli et al. (2018) further published the results of a multi-centric hospital-based study in 2018 to analyze the impact of PCHS® on surface contamination and HAI-related incidence in hospitalized patients in six public hospitals in Italy. They found that cleaning hospital surfaces in six independent public hospitals with PCHS were associated with a significant decrease in HAI cumulative incidence in hospitalized patients (*p* < 0.0001) for 6 months. Concurrently, PCHS® was associated with a stable decrease in investigated surface pathogens (*p* < 0.0001). No infections sustained by PCHS®-derived *Bacillus* spp. were detected in any of the hospitalized patients. Another publication by Caselli et al. (2019) is connected to the above-mentioned study (Caselli et al., 2018) and was conducted in five independent public hospitals in Italy for 6 months. The use of PCHS® was associated with up to 99% decrease in AMR genes, which harbored by surface hospital microbiota. The antimicrobial drug consumption associated with HAI onset showed a 60.3% decrease, with a 75.4% decrease of the associated costs. The results of both studies (Caselli et al., 2018, 2019) were also presented at a conference in 2019 by Rognoni et al. (2019). Caselli and Purificato also published a commentary in 2020 (Caselli and Purificato, 2020) based on both studies (Caselli et al., 2018, 2019) and emphasized that an 83% reduction in surface ESKAPEE pathogens during the PCHS period compared with what was detected during pre-PCHS phase was found. Increase in *Bacillus* spp. microbiota represented approximately 70% of total surface microbiota. PCHS use may provide a novel approach that deserves further exploration.

In 2018, D’Accolti and coauthors published the results of the *in vitro* and *in situ* analysis in a hospital bathroom of the effectiveness of a combined use of phages and PCHS® detergent in removing HAI-related pathogens (D'Accolti et al., 2018). In the *in vitro* assays, the combined use of phages and PCHS® resulted in rapid reduction in the investigated pathogens (*Staphylococcus aureus*, *Escherichia coli*, and *Pseudomonas aeruginosa* strains) and were maintained at low levels, while the effect of phages tended to diminish. In *in situ* assay, the isolated ceramic sink in a hospital bathroom was artificially contaminated with *Staphylococcus aureus* followed by treatment with the combination of phages and PCHS®. The concentration of *Staphylococcus aureus* statistically significantly rapidly declined. D'Accolti et al. (2019) also published another hospital-based study in 2019, analyzing the effectiveness of combined use of phages and PCHS® in reducing staphylococci contamination on hospital surfaces. They found that the daily combined use of targeted phages and PCHS® in bathrooms of general medicine wards in a private hospital in Italy resulted in a rapid and significant reduction in staphylococci load on treated hospital surfaces.

Afinogenova et al. (2018) published the results of a hospital-based study, which was conducted in a treatment room at a Russian medical center using undefined strains of *Bacillus subtilis, Bacillus licheniformis,* and *Bacillus pumilus*. They found that surface treatment with the microbial-based solution resulted in a higher reduction in present pathogens (*Enterococcus faecium*, Enterobacteriaceae, and *Staphylococcus* spp.) compared with the conventionally treated surfaces.

Kleintjes et al. (2019, 2020) published two articles of a hospital-based study in a burn unit of a hospital in South Afrika in 2017 using a cleaning agent that contained unidentified strains of *Bacillus subtilis, Bacillus pumilus,* and *Priestia megaterium*. The first study showed that the microbial-based cleaning agent (August and September 2017) did not significantly reduce the colonization of pathogens for 2 months. The most common organisms isolated were gram-negative bacilli in both arms, such as *Acinetobacter baumannii Enterobacteriaceae*, and *Pseudomonas aeruginosa*. In the latter publication (Kleintjes et al., 2020), an additional month of microbial-based cleaning was conducted in February 2018, and a retrospective analysis of HAI incidence in the hospital was conducted. All months between January 2017 and December 2017, except for the 2 months of microbial-based cleaning, were considered control months. The authors found that the incidence of HAI was statistically significantly lower in the months without microbial-based cleaning. However, the highest increase in the incidence of HAI was noticed right after the first 2 months of microbial-based cleaning, as the HAI incidence in October 2017 was 35. Thus, the long-term effects should be carefully analyzed.

The *in vitro* assessment of the viability Tana® Biotic DC (Tanatex Chemicals, the Netherlands) containing undefined strains of *Bacillus* spores and polyethylene glycol on wipes for surface cleaning was published by Dural-Erem et al. (2019). This study found that it is possible to produce dry wipes that contain an adequate number of beneficial bacteria or spores of Tana® Biotic DC. After wetting, these wipes released a certain number of bacteria which can inhibit pathogens by growing and colonizing on the wiped surfaces. The authors concluded that these wipes could be used for sanitizing in healthcare environments. Razenberg et al. (2020) published a review report in 2020 and discussed the microbial-cleaning products available on the Dutch market. They identified 92 different microbial-cleaning products. However the information on the microbial and chemical composition was often incomplete. It was also unclear to which regulatory framework the products applied. Stone and coauthors published an *in vitro* assay in 2020 (Stone et al., 2020) on the comparison of the effect of surface-cleaning products containing a patented *Bacillus* spore consortium on the resident microbiome. They found that the microbiome established on three surfaces that are common in hospitals (ceramic, linoleum, and stainless steel) by exposure to non-clinical microbiome in a soil science laboratory and an 8-month cleaning period with microbial-based cleaner, followed by inoculation of pathogens, did not completely outcompete the pathogens. However, competitive exclusion was far more effective than disinfectant.

In a multi-centric hospital-based study published by Tarricone et al. (2020), the authors assessed the impact of lower HAI incidence after use of PCHS® compared with conventional chemical cleaning (CCC). This study is also connected with Caselli et al. (2018) and includes the budget impact analysis by comparing the current scenario of the use of CCC with future scenarios and considering increasing utilization of PCHS® in Italian internal medicine, geriatric, and neurology departments. Significant lower incidence of HAI after the use of PCHS compared with CCC and lower incidence of severe HAI and antibiotic resistance could lead to increased health protection and high savings worldwide. In the *in vitro* assay on the antiviral effect of PCHS® published by D’Accolti et al. (2021), it was found that the microbial-based solution PCHS® inactivated 99.99% of all tested enveloped viruses (vaccinia virus, herpesvirus type 1, human alpha coronavirus, human beta-coronavirus, and human and animal influenza viruses) within 1–2 h of contact, both in suspension and on the surface. The antiviral action persisted up to 24 h after application, suggesting that its use may effectively allow a continuous prevention of virus spread via contaminated environment, without worsening environmental pollution and AMR concern. D’Accolti et al. (2022) also published a review in 2022 on the massive use of cleaning and disinfecting of healthcare surfaces in high rates of MDR microbes. They emphasized the importance of stabilizing the built environment microbiome, especially the ‘hospital microbiome’, by remodulation using microbial-based cleaning solutions, such as PCHS®, to lower the concentration of pathogens. The most important pathogens mentioned included *Staphylococcus* spp., *Enterobacteriaceae* spp., *Acinetobacter* spp., *Pseudomonas* spp., *Candida, Aspergillus* spp., and *Clostridioides difficile,* which have been found on hospital surfaces. Reduction in these pathogens on surfaces can result in lowering the incidence of HAIs, MDR pathogens, and HAI-related antimicrobial drug consumption.

Soffritti et al. published a hospital-based study in 2022 (Soffritti et al., 2022) on the assessment of the effect of PCHS® on the emergency room of a children’s hospital in Italy during the COVID-19 pandemic. They found that PCHS® usage on the surfaces in the emergency ward of a childrens’ hospital was associated with a stable 80% decrease in surface pathogens compared with levels detected for chemical disinfection, which was accompanied by an up to 2 log decrease in resistant genes. D’Accolti et al. (2023) also published a new multi-centric hospital-based study in two Italian hospitals on the comparison of the sanitizing effect of microbial-based cleaning using PCHS® with added specific anti-staphylococcal phage. They found that the microbial-based cleaning using PCHS® with added specific anti-staphylococcal phage provided higher removal of staphylococci compared with conventional cleaning. Frequent chlorine usage inactivated the microbial and phage components. Caselli also presented all the latest results on the usage of PCHS® on the surfaces at a conference in 2023 (Caselli, 2023), emphasizing that PCHS® appears to be a sustainable and effective approach to control infectious risk without worsening the pollution and AMR concerns, which are major problems of current times. She also suggests that it could be included as a useful tool in the bundle of actions for effective infection prevention and control strategies.

Klassert et al. published the results of a multi-centric hospital-based study in 2022 (Klassert et al., 2022), which assessed the effect of sanitizing with *Bacillus* spp. containing detergents on the environmental microbiome of a neurological ward in Berlin, Germany. The sanitizing agent contained the following clearly defined strains: *Bacillus subtilis* ATCC 6051, *Priestia megaterium* ATCC 14581, *Bacillus licheniformis* ATCC 12713, *Bacillus amyloliquefaciens* DSL 13563-0, *Bacillus pumilus* ATCC 14884, and surfactants. They found that the environmental microbiota showed a displacement of intrinsic environmental microbiota after cleaning with microbial-based solution in the hospital ward. Statistical significance was reached only in sink samples when compared with traditional disinfection measures (*p* < 0.05). The total number of detected antibiotic resistance genes was statistically significantly different after sanitization with the *Bacillus* spp.-containing cleanser (*p* < 0.01). Another study using the sanitizing product SYNBIO® (HeiQ Chrisal NV) with the exact same strains as above was conducted by Leistner et al. and published in 2023 (Leistner et al., 2023). They found that routine surface disinfection by microbial-based agent SYNBIO® used in non-ICU wards in a tertiary care hospital at four locations in Berlin, Germany, which did not prove to be superior to soap-based cleaning or disinfection in terms of HAI prevention after surveillance of 13,896 admitted patients.

The review by Ramos and Fritz conducted in 2023 (Ramos and Frantz, 2023) on the use of microbial-based solutions in hospital-based studies found that the studies demonstrated overwhelmingly positive results, including significant reductions in pathogen burden, antimicrobial-resistant genes, and nosocomial infections, yet the authors stressed that these studies were limited in duration and scope.

## Cleaning versus sanitization

Numerous authors of the studies in Table 1 employed the term ‘cleaning’ in their research when discussing the reduction in the concentration of pathogens on surfaces using microbial-based products (Vandini et al., 2014a,b; Caselli et al., 2016; Al-Marzooq et al., 2018; Dural-Erem et al., 2019; Kleintjes et al., 2019, 2020; Stone et al., 2020; Leistner et al., 2023). On the other hand, other studies in Table 1 in this context used the term ‘sanitizing’ (La Fauci et al., 2015; Afinogenova et al., 2018; Caselli et al., 2018; Tarricone et al., 2020; Klassert et al., 2022; Soffritti et al., 2022).

According to the CDC (Centre for Disease Control) (CDC, 2023) and EPA (US Environmental Protection Agency) (EPA, 2023), sanitizing specifically aims at reducing microbes, primarily bacteria, to establish a surface that is both safe and has a lower risk of transmitting infectious agents. In contrast, cleaning involves the removal of visible dirt and contaminants. Both procedures contribute to hygiene with the goal of removing, eliminating pathogens from surfaces and usually employ chemicals rather than microbial-based products. Cleaning is more focused on the mechanical removal of dirt containing pathogens, while sanitization is focused on the antagonistic/antimicrobial effect, competitive exclusion effects, and the reduction in the antibiotic resistome of indigenous microbes (Dural-Erem et al., 2019; Rognoni et al., 2019). Both procedures are of course important in the hospital setting and both terms are sometimes used interchangeably (Berihun et al., 2022; Purwandari et al., 2024).

## Is using the term probiotics for microbial-based sanitization appropriate?

The terms ‘probiotics’, ‘probiotic type microorganisms’. or ‘probiotics-based’ are common descriptors of sanitizing formulations discussed in 30 reviewed publications, as shown in Table 1 (Falagas and Makris, 2009; Vandini et al., 2014b; Arvanitakis, 2015; La Fauci et al., 2015; Caselli et al., 2016; Afinogenova et al., 2018; Al-Marzooq et al., 2018, 2019; Caselli et al., 2018; D'Accolti et al., 2018, 2019, 2022, 2023; Rodolfi and Caselli, 2018; Caselli et al., 2019; Dural-Erem et al., 2019; Kleintjes et al., 2019; Rognoni et al., 2019; Caselli and Purificato, 2020; Kleintjes et al., 2020; Razenberg et al., 2020; Stone et al., 2020; Tarricone et al., 2020; Klassert et al., 2022; Soffritti et al., 2022; Caselli, 2023; Leistner et al., 2023; Ramos and Frantz, 2023). Only two publications (Manning et al., 2009; Kohli, 2013) did not use this term.

Although the apparent idea of these authors is to differentiate between pathogenic and beneficial microbes, the use of the term probiotics for non-pathogenic microorganisms is not appropriate. In the reviewed patent, Rodolfi and Caselli (2018) explicitly imply that non-pathogenic bacteria are probiotic, i.e., *per se* beneficial and hence ignoring the commonly used scientific definition of probiotics. Non-pathogenicity alone is not a probiotic qualifier. In our opinion, the term ‘probiotic’ should not be used for microbial cleaners for several reasons.

By definition (Hill et al., 2014), probiotics must be applied to humans or animals (through oral ingestion or application on skin etc.). This definition does not consider inanimate surfaces as application targets because any effect on inanimate surfaces is unrelated to probiotic health claims. Moreover, there is no reference in the reviewed studies that the bacterial components are used or authorized as probiotics for humans or animals. The term probiotic is commonly used for food or dietary supplements in some cases for skin application or other topical applications. In the European Union probiotics, dietary supplements or feed additives are subjected to pre-market quality, safety, and efficacy assessment by EFSA prior to market authorization. Live microorganisms, i.e., probiotics for medicinal use are regulated and authorized by the European Medicinal Agency or FDA. These agencies scrutinize strain level characteristics and related potential risks to humans, animals, and environment along with occupational safety and product quality. It was the aim of several studies to reduce AMR. Therefore, it is prudent and appropriate to provide evidence that the microbial components of the tested products do not carry genetic elements of AMR which may contribute to the increase in the antibiotic resistome in hospitals. Using the term probiotic in relation to microbial-based cleaning products strongly implies that similar safety, quality, and efficacy assessments took place prior to commercialization. Since most product labels do not state strain identity, it is reasonable to assume that safety has not been established. It is also not correct to generalize safety assumptions to an entire species and label a whole species as probiotic because of the intra-species genetic variability. Therefore, it is only prudent for future researchers and hospital management to require third party strain level safety assessment of microbial-cleaning products before introducing hospital use.

Nevertheless, there are numerous probiotic strains of various *Bacillus* spp. used as dietary supplements with proven health benefits, as evidenced in randomized controlled clinical trials. Such studies investigate the effectiveness of probiotic administration interventions after carefully selecting patients and outcomes produced by intention-to-treat analysis (Hariton and Locascio, 2018). For example, *Bacillus subtilis* BS50 decreased gastrointestinal symptoms in healthy human adults (Garvey et al., 2022), *Heyndrickxia* (previously *Weizmannia* and before that *Bacillus*) *coagulans* MY01 and *Bacillus subtilis* MY02 decreased functional dyspepsia in human adults (Wauters et al., 2021), *Heyndrickxia coagulans* Unique IS2 significantly decreased the symptoms of constipation in human adults (Madempudi et al., 2020), a mixture containing *Bacillus subtilis* DE111, *Priestia megaterium* MIT411, *Heyndrickxia coagulans* CGI314, and *Bacillus clausii* CSI08 improved loose stools and proved safe without negative changes in adults. Strains of *Bacillus amyloliquefaciens* have been investigated in animal trials (González-Ortiz et al., 2013; Ahmed et al., 2014; Rea et al., 2023). Probiotics in quality clinical trials are always identified on strain level, acknowledging the fact that health effects are strain-specific as also emphasized in the consensus statement of the International Scientific Association for Probiotics and Prebiotics (Hill et al., 2014). According to Binda et al. (2020), for a strain to be labeled as probiotic, a statistically significant health benefit must be evidenced in at least one placebo controlled clinical trial. However, the probiotic health benefits of a food, dietary supplement, or feed additives cannot be extrapolated to the use on inanimate surfaces since the purpose of cleaning application is not the ingestion of microbes. Therefore, it is inevitable to underline that calling microbial-cleaning products for probiotic is inappropriate and misleading. The probiotic term is used broadly for food chain-related products, such as fermented dairy products, dietary supplements, and feed additives. Using the term probiotic for a cleaning or sanitizing agent may be misleading and could even encourage intentional ingestion of the product, with possible harmful consequences caused by the microbial agents or non-food chemical components. This intrinsic hazard needs further considerations when product safety and labeling are in question.

## Assessment of safety and antimicrobial resistance (AMR) of microbes in microbial-based sanitizing agents

In microbial sanitizing, the absence of mobile genetic elements related to transferable antimicrobial resistance (AMR) is a minimum requirement, considering that the intention of use is to limit the spread of AMR. The safety of using microbial-based sanitization in this context means that the strains applied on surfaces could also potentially reach the patients. Hence, the questions are as follows: Do these strains produce risk factors, e.g., toxins and antibiotics? Do they contain mobile, i.e., transferable genetic elements of anti-microbial resistance? Some authors addressed some of these aspects.

Klassert et al. (2022) assessed AMR and found that microbial-based sanitization did not statistically significantly decrease the prevalence of antibiotic resistance genes for mecA compared with conventional methods, except for the sink samples. The authors tested only a few antimicrobial resistance genes (ARGs) and acknowledged this limitation. Vandini et al. (2014b) assessed the susceptibility and AMR of the microbial components in PCHS® against five antibiotics, namely, penicillin, cefoperazone, cefalotin, gentamicin, and clindamycin. As to the product, all *Bacillus* spp. were resistant to penicillin and showed intermediate resistance to cefoperazone and clindamycin. However, this scope of antibiotics did not comply with relevant EFSA guidelines on the assessment of bacterial susceptibility to antimicrobials of human and veterinary importance (EFSA, 2012). According to these guidelines, the minimum inhibitory concentration of the following antimicrobials should be determined: ampicillin, chloramphenicol, clindamycin, erythromycin, gentamicin, kanamycin, streptomycin, tetracycline, and vancomycin. Among the latter, only two antibiotics overlap with those tested by Vandini et al. (2014b). Establishing the relevant antibiotic resistance profile of PCHS®^−^related *Bacillus* spp. on strain level demands further investigation.

For the assessment of the presence of potentially transferable genetic elements of AMR in the product, Vandini et al. (2014b) also compared the AMR profile of a reference strain with the *Bacillus* spp. component of PCHS®. The objective of this generally acknowledged approach is to identify AMR that is intrinsic to the whole bacterial species and can be considered as non-transferable and hence non-hazardous. However, the applied reference strain *Bacillus subtilis* ATCC 6633, which has been since reclassified as *Bacillus spizizenii* ATCC 6633 (Dunlap et al., 2020), is a different species. This implies that the conclusion on mainly intrinsic AMR was based on the comparison between different species. Therefore, further investigation between the same species is needed to affirm the original conclusion. As to intrinsic AMR, Vandini et al. (2014b) compared the reference strain of *Bacillus spizizenii* ATCC 6633 (former *Bacillus subtilis*) with the undefined *Bacillus* consortium of PCHS®. In the absence of strain level identity of the *Bacillus* consortium of the product, strain level comparison was obviously not feasible though desirable. Additional antibiotic resistance genes were also detected by qPCR microarray against aminoglycosides, chinolone, and macrolides. After 12 months of use, no change in the resistome of the hospital’s indigenous *Bacillus* population could be detected. The effect on non-*Bacillus* spp. resistome was not investigated.

Caselli (2023) concluded that no acquisition of new genes was observed in the PCHS product despite the continuous contact with virulent and resistant pathogens, and no infections were detected ascribable to PCHS during more than 10 years of application. Tarricone et al. (2020) found that in PCHS, *Bacillus* spp. were shown to contain a few chromosomal not-transferable resistance genes, and the gene exchange between them and the surrounding pathogens was assessed in each treated structure in over 600 *Bacillus* isolates from treated surfaces. No acquisition of new resistance genes was detected in any *Bacillus* spp. sample, supporting their high genetic stability despite continuous contact with resistant pathogens. They also found that gene exchange mechanisms were not favored on hard dry inanimate surfaces. Notably, the dissemination of AMR to non-*Bacillus* spp. was not investigated in these studies.

Three studies (D'Accolti et al., 2018, 2019, 2023) also included phages that infect bacteria, as an emerging alternative to conventional antibiotics usage. However, phages are known as potential vectors of horizontal AMR transfer; hence if a product contains phages as an active agent, the safety assessment must be extended to possible AMR-carrying genetic elements in the genetic material of the phage (Pfeifer et al., 2022).

Due to the absence of information on strain identity, third party risk assessment of microbial cleaners is impossible. Such situation leaves hospitals and patients at risk of using potentially hazardous agents.

## Possible modes of action of microbial-based sanitizing agents

The hypothesis of adding beneficial microbes to hospital environmental surfaces is based on the assumption that we cannot keep these surfaces free of microbes and should therefore utilize non-pathogenic microorganisms and their metabolites to prevent colonization and biofilm formation of pathogens (Falagas and Makris, 2009). This hypothesis implies that the microbes applied are viable at the time of application and can grow on cleaned hospital surfaces. In this regard, the following questions arise: Do *Bacillus* spores germinate into metabolically active vegetative cells on nutrient depleted, desiccated hard surfaces in hospitals? If so, what induces their germination and what kind of germinants or trigger compounds are in the sanitizing formulations to facilitate germination? Can these components also promote the germination of environmental spores? Furthermore, what nutrients are required to keep vegetative cells alive, and would these nutrients also promote the growth of contaminant microbes? In some of the reviewed studies, these questions are addressed marginally.

Several studies (Vandini et al., 2014b; Caselli et al., 2019; Stone et al., 2020; Klassert et al., 2022) indicate the significant role of biofilm formation in constraining the proliferation of pathogenic microorganisms, particularly in healthcare environments. Biofilms, formed from germinating spores into vegetative cells, create stable structures that inhibit pathogen growth and compete with pathogens on treated surfaces, which is the process of competitive exclusion. Dural-Erem et al. (2019) employed moisturized wipes (Tana® Biotic DC) to evenly spread bacteria or spores onto inanimate surfaces. The released bacteria inhibited pathogens by hypothesized competitive exclusion, hence ensuring sanitizing effect on healthcare environments. However, the contribution of other components such as detergents or microbial metabolites to this effect was not specifically investigated in their study. Understanding the relative contributions of these components—bacteria, spores, detergents, and microbial metabolites—could provide valuable insights into optimizing sanitization protocols, particularly in healthcare settings where controlling pathogen spread is crucial. Further research on the individual and combined effects of these components could help refine strategies for preventing infections and promoting hygiene.

Overall, 4–5% of the genome of *Bacillus* spp. encodes antimicrobial compounds. These compounds have substantial potential to enhance the sanitizing effect. For example, *Bacillus subtilis* is able to produce more than two dozen antibiotics and other antimicrobial compounds (Stein, 2005). Other *Bacillus* spp., such as the recently renamed *Heyndrickxia coagulans* PL-W (previously *Bacillus coagulans*) (Gupta et al., 2020; Narsing Rao et al., 2023), produce various bacteriocins (Wang et al., 2023). *Bacillus amyloliquefaciens* and other *Bacillus* spp. produce biosurfactant lipopeptides such as surfactins, iturins, and fengycins with antifungal antibacterial and antiviral properties (Caulier et al., 2019; Englerová et al., 2021). *Bacillus* spp. also produce a large number of antibiotics, i.e., bacitracin, bacilysocin, and bacimethrin, to name a few (Nakano and Zuber, 1990), which act against gram-negative and gram-positive pathogens. Consequently, it is reasonable to assume that bioactive metabolites of *Bacillus* spp. also contribute to the sanitizing effect. Microbial-cleaning products also contain preservatives to prevent the growth of contaminant microbes. The concentration and effect of the latter are not discussed in any of the reviewed studies.

Most of the reviewed studies emphasize competitive exclusion as the main mechanism of action without considering antimicrobial production. Competitive exclusion describes both the process and the result of microbial competition for space or nutrients when co-occurring bacterial species in the same ecological niche compete for limited resources. This competitive strategy is two-fold: exploitation and interference. Exploitation competition is indirect, which is characterized by rapid resource consumption (restricting supply to competitors and investing in growth), whereas interference competition occurs when one organism directly harms another one through the production of antimicrobial compounds (Knipe et al., 2020). Applying bacteria that are capable of producing antimicrobial compounds to eliminate competitors is a questionable strategy when the overall objective is to decrease AMR resistome. Antimicrobial production may trigger defensive adaptations, i.e., the emergence of new AMR genes that consequently enlarge the environmental resistome in hospitals. This outcome would be highly undesirable considering the overall objective when using microbial-cleaning products in hospitals is to reduce AMR resistance. Applying well characterized bacterial strains without antibiotic, or antimicrobial production capabilities, would reduce the competitive exclusion mechanism to the harmless competition for nutrients and space. This requires further research to prove whether such approach would be sufficiently effective.

Conventional detergents, surfactants, disinfectants, and preservatives are usually part of the base solution of microbial-cleaning products (Klassert et al., 2022). The non-microbial components may also substantially contribute to the antibacterial effect (Manning et al., 2009; La Fauci et al., 2015).

## HAIs incidence in hospital settings after use of microbial-based sanitizing agents

Numerous studies have highlighted the efficacy of microbial-based sanitization products in reducing the presence of common pathogens on hospital surfaces. Among these pathogens are *Enterococcus faecalis, Pseudomonas aeruginosa*, *Acinetobacter baumannii, Klebsiella pneumoniae, Staphylococcus aureus,* and *Candida albicans*. In a series of investigations cited (La Fauci et al., 2015; Afinogenova et al., 2018; Al-Marzooq et al., 2018; D'Accolti et al., 2018; Caselli and Purificato, 2020), the prevalence of these pathogens notably decreased following the application of such products. Furthermore, the use of microbial-based sanitization correlated with a decrease in hospital-acquired infections (HAIs) among patients, subsequently leading to a reduction in antimicrobial resistance (AMR) and a decreased reliance on antibiotics (Caselli et al., 2019; Rognoni et al., 2019; D’Accolti et al., 2022). However, contrasting findings emerged in studies conducted by Leistner et al. (2023), Kleintjes et al. (2019), and Leistner et al. (2023) reported that the microbial-based product SYNBIO® did not exhibit superiority over conventional soap-based cleaning in preventing HAIs, while Kleintjes et al. (2019) found that a microbial-based cleaning agent containing unidentified strains of *Bacillus subtilis, Bacillus pumilus*, and *Priestia megaterium* did not significantly reduce pathogen colonization. It is plausible that discrepancies in effectiveness stem from variations in microbial strains, dosages, and duration of sanitization protocols across studies. These findings underscore the importance of meticulous attention to such parameters in maximizing the efficacy of microbial-based sanitization strategies.

## Microbial cleaning and biocide regulation

The European Union initially Directive 98/8/EC3 regulated biocidal products on a community level (EUR-Lex, 1998). In 2012, it was replaced by EU regulation 528/2012 (EUR-Lex, 2012), and its consolidated version is in force since 2022 (EUR-Lex, 2022). Effectiveness, quality, safety to the human’s environment, and occupational safety of the active agents are to be assessed prior to placing on the market. Following positive assessment, active substances can be used in commercial formulations. It appeared for a considerable period that microbial-based cleaning products did not fit into the existing legislation of neither detergents nor biocides. In the absence of enforced regulatory guidelines, products without safety and efficacy assessment (Razenberg et al., 2020) have been broadly commercialized. A few years ago, a relevant ruling of the Court of Justice of the European Union ultimately clarified the status of the category (EUR-Lex, 2019). The court concluded that the effect of live microbes on harmful microorganisms is a biocidal effect regardless of the mode of action and affirmed that the product in question should be classified as an unauthorized biocidal product and hence may not be placed on the EU market. In the Courts, interpretation products containing one or more bacterial species are biocidal products even if not having a direct effect on harmful organisms. Notably, the product at issue was explicitly named ‘probiotic cleaning product’ containing a consortium of *Bacillus* spp. Hence, cleaning products containing one or more bacterial species are biocidal products and hence require authorization prior to placing on the market of the EU.

Currently, five *Bacillus* strains are approved as biocidal active substances as listed in the European Chemical Agency database (ECHA) for use in the European Economic Area and Switzerland. *Bacillus amyloliquefaciens* ISB06 is approved as a sanitizing agent for veterinary hygiene purposes (ECHA/BPC/085/2015, 2015; ECHACHEM, 2018). The other four strains (*Lysinibacillus sphaericus* ABTS-1743, *Bacillus thuringiensis* subsp. *israelensis* Serotype H14 AM65-52, *Bacillus thuringiensis* subsp. *israelensis* SA3A, and *Bacillus thuringiensis* subsp. *kurstaki* ABTS-351) are approved for controlling insects and ants (ECHACHEM, 2013, 2016a,b, 2017). The microbial components that were used in the reviewed studies noted in Table 1 are not listed in the ECHA database of biocidal agents.

Considering that the legal framework of the European Union exists, it remains a major concern that none of the microbial based products investigated in European hospital settings is authorized. Further investigation is required to understand the reasons for this situation.

## Addressing gaps and future perspectives of microbial cleaning

Microbial cleaning is a popular segment of bioremediation, which is a safe and environmentally friendly use of microbes to accelerate the breakdown of organic compounds (Kohli, 2013). Although microbes have the potential to sanitize indoor environments, their use in hospitals requires particular considerations and additional research, (La Fauci et al., 2015) such as antimicrobial resistance, long-term survival, strain sequencing, and product safety (Falagas and Makris, 2009). Such information is crucial in hospital settings. The potential of certain microbes is effective in eliminating pathogens to reduce the genetic pool of antibiotic resistance in hospitals. Microorganisms, used for microbial cleaning, must be non-pathogenic and classified as class I (biosafety level I), which do not require special biosafety facilities for handling and use (Kohli, 2013). Classification at the strain level is necessary since even species that are considered generally harmless can harbor strains with pathogenic potential. For example, although the majority of *Bacillus subtilis* strains exhibit minimal virulence, they can also be causative pathogens (Lampropoulos et al., 2021). Since patients in hospitals are particularly vulnerable and many times immunocompromised, their safety must be considered before opting for novel sanitizing regimes. Microbial-cleaning products may pose a risk to humans as possible causative agents of infection, intoxication, irritation, hypersensitivity reaction, contact allergy, or through the dissemination of antibiotic resistance genes or undesired antimicrobial compounds (Razenberg et al., 2020). Therefore, safety assessment before placing on the market is necessary. Currently, microbial-cleaning products are subjected to the Biocidal Product Regulation (ECHA, 2023) of the European Union. Remarkably, though none of the microbial components used in the reviewed studies is authorized for use and hence cannot be considered as safe. Further investigations on whether spores germinate and become metabolically active after distribution on fast-drying, nutrient-depleted hard surfaces in hospital settings are also crucial.

The current research on microbial-based cleaning shows promise, advocates for reducing our dependence on chemical disinfectants, and promotes the adoption of holistic and sustainable sanitation methods that effectively eliminate pathogens while preserving a beneficial microbiome in our built environments. However, before the potential widespread use of microbial-based cleaning, it is important to address the remaining uncertainties and challenges in this field (Ramos and Frantz, 2023).

## Conclusion

In conclusion, this review emphasizes that microbial-based cleaning is an interesting concept with the potential to control hospital-acquired infections. Most products reviewed did not disclose the strain identity of the applied bacteria. Without strain level identification, safety assessment of the bacterial components is impossible. Consequently, microbial-based cleaning products may pose a risk to hospital patients and personnel. Although the existing biocide regulatory frameworks provide an adequate safety assessment platform, none of the microbial-based cleaning products reviewed in this article underwent safety assessment within this framework. Therefore, precise and correct up-to-date characterization of microbial agents in cleaning products is necessary, particularly if such products are to be used in hospitals. More robust, well-designed trials with properly characterized microbial components and defined mode of action are required. Only the use of authorized microbial components in hospital settings should be best practiced for future research of the category.

## Author contributions

SF: Writing – review & editing, Writing – original draft, Supervision, Methodology, Investigation, Data curation, Conceptualization. PK: Writing – review & editing, Writing – original draft. UR: Writing – review & editing. SŠ: Writing – review & editing.
